# Whole-exome sequencing reveals genetic variants in ERC1 and KCNG4 associated with complete hydatidiform mole in Chinese Han women

**DOI:** 10.18632/oncotarget.20769

**Published:** 2017-09-08

**Authors:** Yan Yu, Bingjian Lu, Weiguo Lu, Shuang Li, Xiuqin Li, Xinyu Wang, Xiaoyun Wan, Yaxia Chen, Suwen Feng, Yao Jia, Ru Yang, Fangxu Tang, Xiong Li, Shulan Zhang, Xinyan Wang, Heng Wei, Zhilan Peng, Lin Lu, Huizhen Zhong, Linjun Zhao, Zhangqian Huang, Lin Lin, Weihong Shen, Yan Lu, Zhu Cao, Jian Zou, Yuejiang Ma, Xiaojing Chen, Qifang Tian, Shiming Lu, Pengyuan Liu, Ding Ma, Xing Xie, Xiaodong Cheng

**Affiliations:** ^1^ Key Laboratory of Women’s Reproductive Health of Zhejiang Province, Women’s Hospital School of Medicine, Zhejiang University, Hangzhou, Zhejiang, China; ^2^ Department of Surgical Pathology, Women’s Hospital School of Medicine, Zhejiang University, Hangzhou, Zhejiang, China; ^3^ Department of Gynecologic Oncology, Women’s Hospital School of Medicine, Zhejiang University, Hangzhou, Zhejiang, China; ^4^ Department of Obstetrics and Gynecology, Tongji Hospital, Tongji Medical College, Huazhong University of Science and Technology, Wuhan, Hubei, China; ^5^ Department of Obstetrics and Gynecology, Shengjing Hospital of China Medical University, Shenyang, Liaoning, China; ^6^ Department of Obstetrics and Gynecology, West China Second Hospital of Sichuan University, Chengdu, Sichuan, China; ^7^ Department of Obstetrics and Gynecology, Ningbo Women and Children’s Hospital, Ningbo, Zhejiang, China; ^8^ Department of Obstetrics and Gynecology, Shaoxing Women and Children Hospital, Shaoxing, Zhejiang, China; ^9^ Zhejiang University Hospital, Zhejiang University, Hangzhou, Zhejiang, China; ^10^ Institute for Translational Medicine School of Medicine, Zhejiang University, Hangzhou, Zhejiang, China; ^11^ Department of Clinical Laboratory, Women’s Hospital School of Medicine, Zhejiang University, Hangzhou, Zhejiang, China; ^12^ Sir Run Run Shaw Hospital School of Medicine, Zhejiang University, Hangzhou, Zhejiang, China

**Keywords:** genomics, whole-exome sequencing, complete hydatidiform mole, pathogenesis

## Abstract

Complete hydatidiform mole (CHM) is a rare pregnancy-related disease with invasive potential. The genetics underlying the sporadic form of CHM have not been addressed previously, but maternal genetic variants may be involved in biparental CHM. We performed whole-exome sequencing of 51 patients with CHM and 47 healthy women to identify genetic variants associated with CHM. In addition, candidate variants were analyzed using single base extension and Matrix Assisted Laser Desorption/Ionization-Time of Flight Mass Spectrometry in 199 CHM patients and 400 healthy controls. We validated candidate variants using Sanger sequencing in 250 cases and 652 controls, including 205 new controls. Two single nucleotide polymorphisms, c.G48C(p.Q16H) in*ERC1* and c.G1114A(p.G372S) in *KCNG4*, were associated with an increased risk of CHM (*p*<0.05). These variants may contribute to the pathogenesis of CHM and could be used to screen pregnant women for this genetic abnormality.

## INTRODUCTION

Complete hydatidiform mole (CHM) is a rare pregnancy-associated disease that can spread to distant sites [1]. Women with CHM have a 1,000-fold increased risk of gestational trophoblastic neoplasia (clinically aggressive lesions consisting of choriocarcinoma, placental site trophoblastic tumor, and epithelioid trophoblastic tumor), compared to women who have had a term pregnancy [2]. Hydatidiform mole exhibits an unbalanced geographic distribution [3-7]. The incidence is highest in Southeast Asia (3.89–13.99 per 1,000 pregnancies) and lowest in Latin America, North America, Europe, and Oceania (0.23–1.21 per 1,000 pregnancies). The incidence of CHM in Chinese women is approximately five per 1,000 pregnancies [6].

Cytogenetic and molecular pathology data indicate CHM has an androgenetic origin [8, 9]. Lack of maternal genomic imprinting also plays an important role in CHM [10]. Causal mutations in the maternal genes *NLRP7*(Nacht Domain-, Leucine-rich Repeat-, and PYD-containing protein 7)[11-13] and *KHDC3L*/*C6orf221*(KH domain containing 3 like) have been identified in women with familial, recurrent bi-parental CHM, which accounts for approximately 20% of all CHM [11]. Women with a history of CHM is have a 5–40 times higher relative risk of recurrent CHM than other women, regardless of changes in sexual partner. Thus, maternal genetic rather than environmental factors may play a predominant role in CHM.

**Figure 1:**
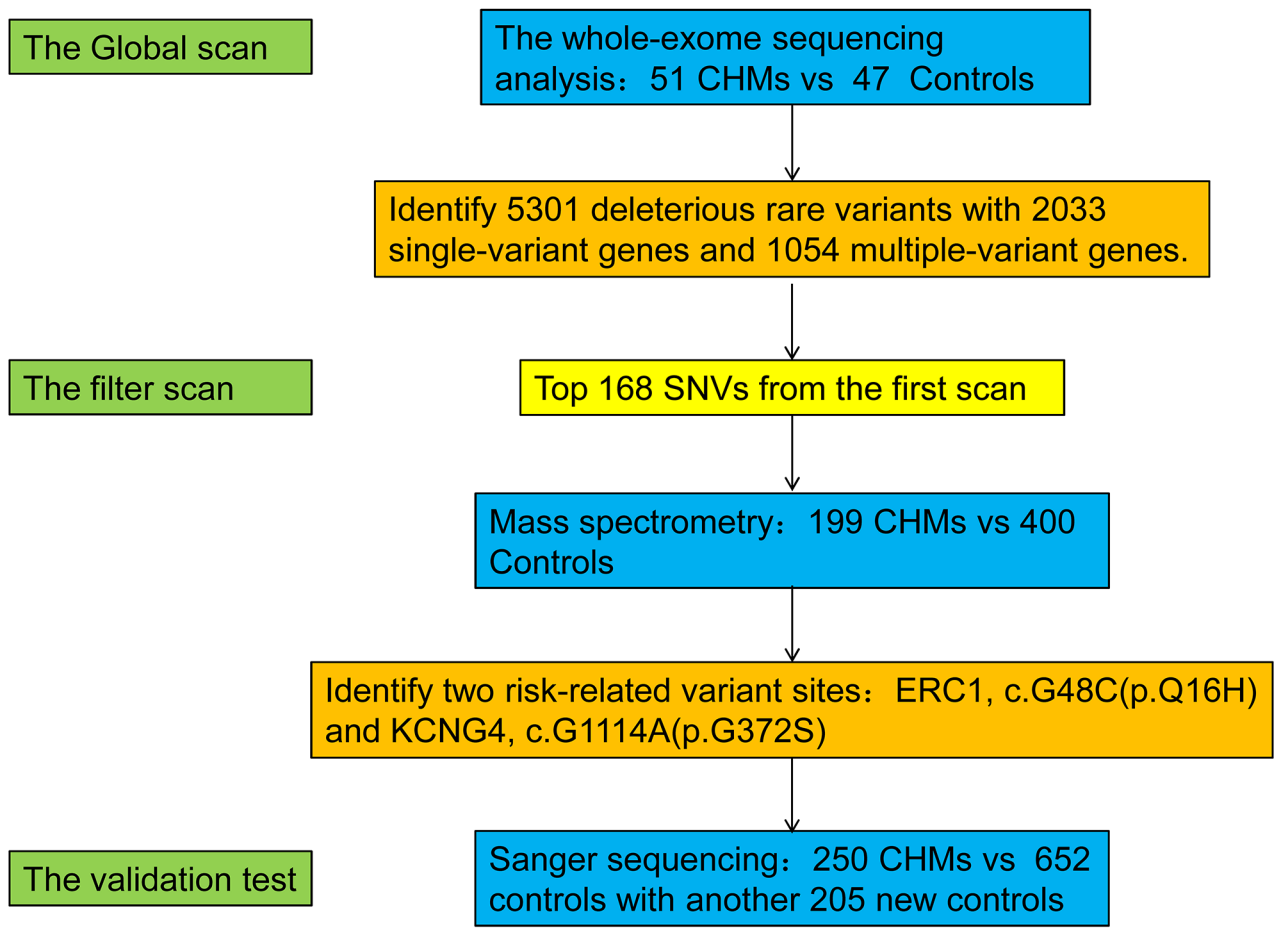
Summary of the study design and results We performed whole-exome sequencing on samples from 51 CHM patients and 47 healthy women. Screening of candidate mutations by MALDI-TOF MS was performed in 199 CHM patients and 400 healthy women. Candidate polymorphisms were validated by Sanger sequencing in 250 cases and 652 controls, which included samples analyzed in the first and second screening and an additional 205 controls. We identified two SNPs:*ERC1*c.G48C(p.Q16H), and *KCNG4* c.G1114A(p.G372S) that were associated with an increased risk of CHM (p<0.05).

Next-generation sequencing is a fast and cost-effective method for generating genome-scale sequencing data and has contributed to the search for disease- and trait-related genetic variants [14, 15]. Whole-exome sequencing allows the discovery of low-frequency variants in individuals with familial, highly penetrant diseases, and those with complex quantitative traits [16-19]. We performed whole-exome sequencing to identify genetic variants that contribute to non-familial CHM in Chinese Han women. Candidate variants were further analyzed by single base extension (SBE) and Matrix Assisted Laser Desorption/Ionization-Time of Flight Mass Spectrometry (MALDI-TOF MS)[20]. An overview of the experimental workflow is shown in Figure 1.

## RESULTS

### Whole-exome sequencing analysis

**Figure 2:**
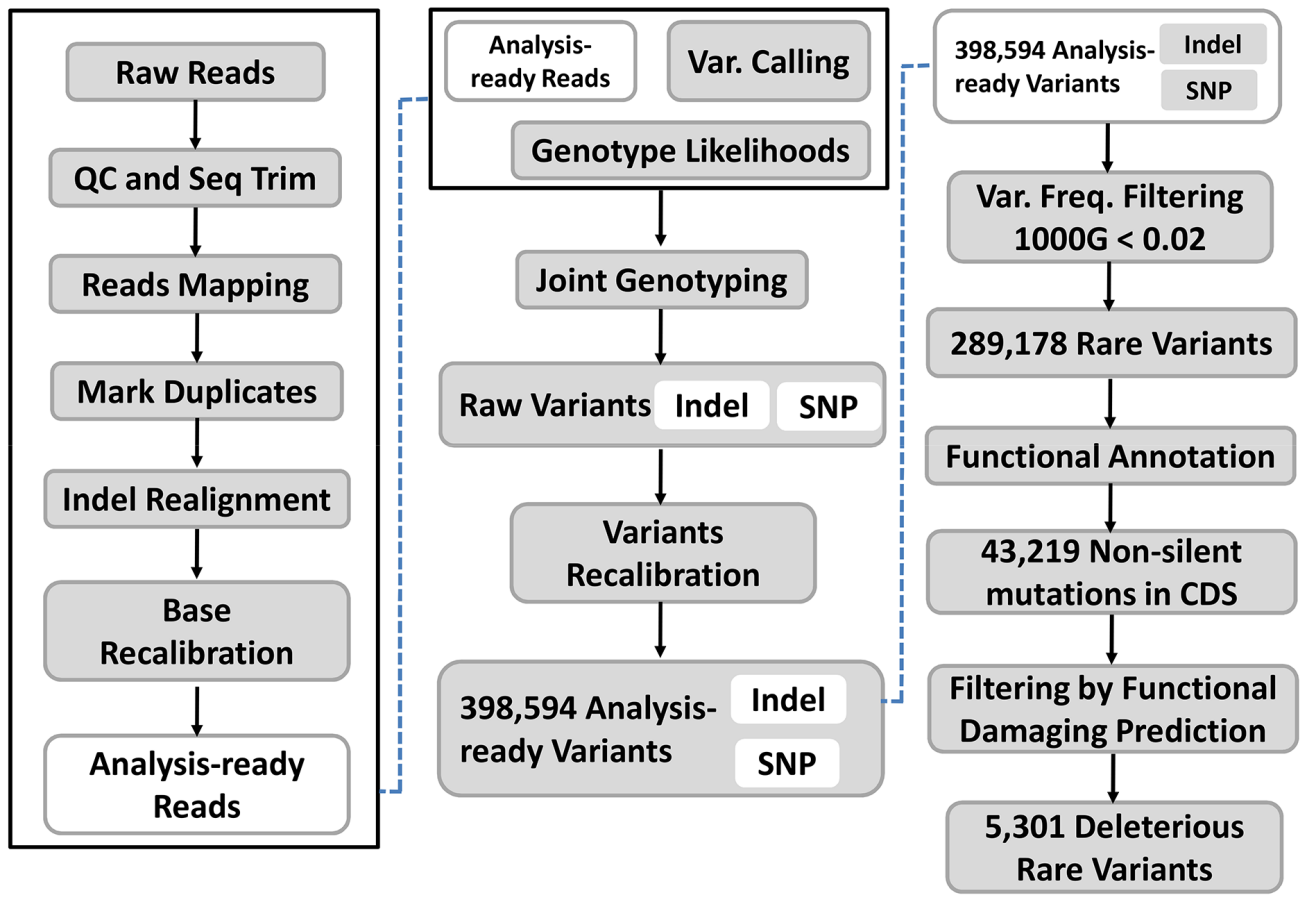
Analysis of whole-exome sequencing data We identified 398,594 SNVs. There were 289,178 rare SNVs in 2,034 genes (MAF<0.02, in the 1000 genomes project (http://1000genomes.org)) and 43,219 non-silent mutations in CDS regions. Functional analysis indicated there were 5,301 rare variants predicted to be deleterious. MAF for each SNV was calculated according to the guidline from https://www.ncbi.nlm.nih.gov/projects/SNP/docs/rs_attributes.html.

**Figure 3:**
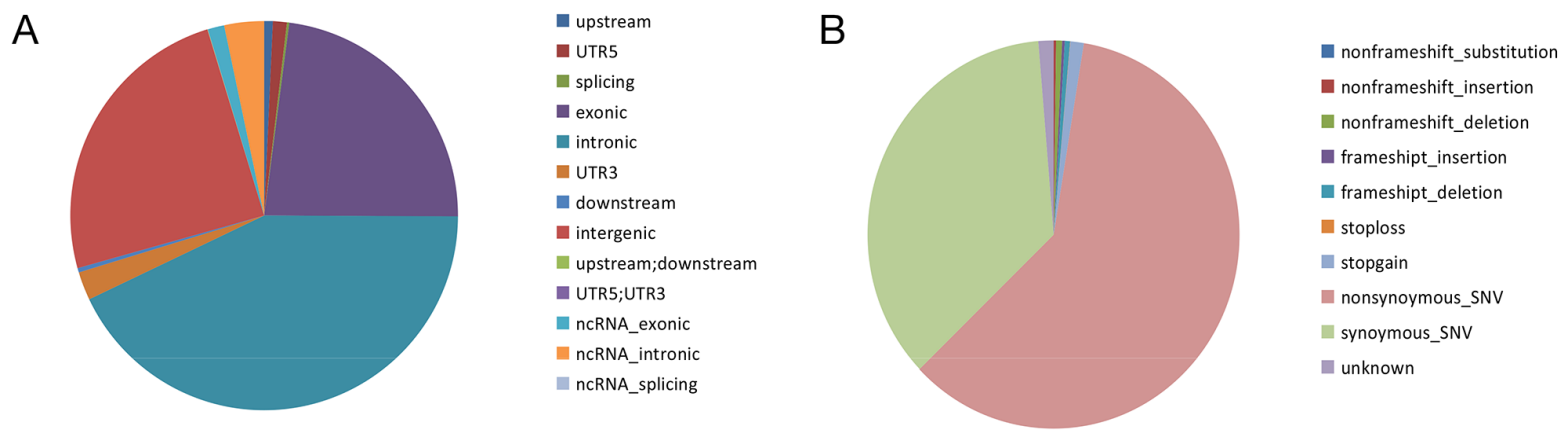
Distribution and functional analysisof rare SNVs **(A)** Of the 289,178 rare SNVs identified, there were 2,035 SNVs in upstream regions; 3,367 SNVs in 5’UTRs; 560 SNVs in splice sites; 66,536 SNVs in exons; 123,938 SNVs in introns; 6,776 SNVs in 3’UTR; 1,085 SNVs in downstream regions; 71,210 SNVs in intergenic regions; 117 SNVs in upstream/downstream regions; 6 SNVs in 5’UTR/3’UTRs; 3,923 SNVs in ncRNA exons; 9,591 SNVs in ncRNA introns; 34 SNVs in ncRNA splice sites. **(B)** Functional analysis of the 43,219 SNVs inCDS regions revealed 7 SNVs that resulted in non-frameshift substitutions; 127 SNVs that resulted in non-frameshift insertions; 356 SNVs that resulted in non-frameshift deletions; 142 SNVs that resulted in frameshift insertions; 306 SNVs that resulted in frameshift deletions; 26 stop-loss SNVs; 764 stop-gain SNVs; 40,064 non-synonymous SNVs; 23,877 synonymous SNVs; and 867 SNVs of unknown consequence.

The initial screening set included 98 samples (51 CHM and 47 controls). On average, we generated 72,135,990 high-quality reads per sample to a mean depth of 67-fold exon coverage (Supplementary Table 2). We identified 398,594 candidate variants (Figure 2). Most were single nucleotide variants (SNVs). Single nucleotide substitutions or inversions were the predominant types of non-synonymous variants. The major types of nucleotide alterations were C>T and G>A transitions. These SNVs were first filtered using the 1000 genome project with a minor allele frequency (MAF) less than 0.02. After filtering, these rare variants were subject to annotation using the NCBI RefGene database (hg19). A total of 43,219 SNVs were non-silent mutations in coding sequence (CDS) regions(Figure 3A). We then used multiple software tools, including SIFT, PolyPhen, LRT, MutationTaster, MutationAssessor, FATHMM, MetaSVM, and MetaLR, to predict whether the non-silent variants affected protein function [21]. Deleterious variants were defined as those predicted to affect protein function by at least five of the above tools. As a result, 5,301 rare variants were predicted to be deleterious in 2,033 single-variant genes and 1,054 multiple-variant genes(Figure 3B). Candidate SNVs are shown in Supplementary Table 3.

### Mass spectrometry analysis

Fisher’s exact tests were used to test the association between the aforementioned 2,033 single-variant genes and CHM risk. The top 41 genes containing 41 variants were chosen for further validation. AssotesteR analysis was used to evaluate the association between the aforementioned 1,054 multiple-variant genes and CHM risk. The top 46 genes containing 127 variants were chosen for further validation. The top 168 rare SNVs were chosen from the 5,301 deleterious rare variants for MALDI-TOF mass spectrometry analysis. We hypothesized that these rare SNVs were the most likely to be associated with CHM. Four SNVs were excluded due to the difficulty of primer design or failures due to simultaneous detection of other loci. Thus, 164 SNVs were detected in 6 wells in the mass spectrometry analysis of samples from 199 new CHM patients and 400 new normal controls (Supplementary Table 4). Using mass spectrometry analysis, the minor alleles of 41 SNVs were not detected in either CHM cases or healthy controls. All sites except two were in Hardy-Weinberg equilibrium. Therefore, 121 SNVs were retained for subsequent association analysis. Two variants, *ERC1*c.G48C (p.Q16H) (p=0.013) and *KCNG4* c.G1114A(p.G372S)(p = 0.014), were associated with an increased risk of CHM. Several other SNVs in *SDK2*, *KIAA1462*, *ZNF799*, *Cllorf21*, and *BBS9* were also associated with CHM risk (Supplementary Table 5).

### Identification of candidate variants associated with CHM risk

We sequenced two sites, *ERC1* c.G48C(p.Q16H) and *KCNG4* c.G1114A(p.G372S) in 902 samples (250 CHM and 652 controls, including 205 new controls). Three CHM cases were excluded because the genomic DNA was not available at this stage. In this validation set, 98 subjects (51 CHM cases and 47 controls) were sequenced using both exome sequencing and Sanger methods. The SNV results of these two sites showed 100% concordance between the two sequencing methods. Similarly, the comparison between mass spectrometry and direct sequencing showed 99.8% concordance between these two sites. High concordance was also observed among the three methods (R^2^=0.9999) (Supplementary Table 6). *ERC1* c.G48C(p.Q16H) was associated with an increased risk of CHM in all samples (p<0.01, odds ratio [OR]=2.69, Table 1), and there were 20 patients have this variant. *KCNG4* c.G1114A(p.G372S) was also associated with an increased risk of CHM (*p*<0.01, OR=2.68, Table 2), and there were 13 patients with this variant, including one patient homozygous for the variant.

**Table 1:** Analysis of the association between *ERC1*(c.G48C) and CHM

ERC1(c.G48C)	CHM patients		Controls		p	OR (95% CI)
	n*	RAF/ALL	n*	RAF/ALL		
WES	51(51)	5/102	47(47)	2/94	0.26	
MassARRAY	198(199)	15/396	399(400)	12/798	0.013	2.58 (1.26 - +∞)
Sanger test	248(250)	20/496	651 (652)	20/1302	0.002	2.69(1.51 - +∞)

RAF=risk allele frequency; n=number.

* The number given in bracket represents for the total of samples for testing while that outside of the bracket indicates the total of the samples with interpretable results. Several cases failed in these detection systems.

**Table 2:** Analysis of the association between *KCNG4*(c.G48C) and CHM

KCNG4(c.G48C)	CHM patients		Controls		p	OR (95% CI)
	n*	RAF/ALL	n*	RAF/ALL		
WES	51(51)	3/102	47(47)	0/94	0.14	
MassARRAY	197(199)	11/394	395(400)	7/790	0.014	3.21(1.31 - +∞)
Sanger test	247(250)	14/494	650 (652)	14/1300	0.009	2.68(1.33 - +∞)

RAF=risk allele frequency; n=number.

* The number given in bracket represents for the total of samples for testing while that outside of the bracket indicates the total of the samples with interpretable results. Several cases failed in these detection systems.

## DISCUSSION

We identified genetic variants associated with CHM risk in a Chinese Han population using whole-exome sequencing. Variants were validated using mass spectrometry and Sanger sequencing. Previous studies have demonstrated that exome sequencing enables the discovery of rare variants associated with various cancers, heritable diseases, and complex quantitative traits [16-19]. We have demonstrated that whole-exome sequencing can be used to screen patients for rare disease-associated germline variants.

We identified two new variants,*ERC1*c.G48C(p.Q16H) and *KCNG4* c.G1114A(p.G372S), that were associated with risk of CHM in Chinese women.ERC1 is a RIM-binding protein. RIMs are active zone proteins that regulate presynaptic neurotransmitter release. Immunocytochemical analysis and live cell imaging showed that YFP-conjugated ELKS translocated to the plasma membrane after antigen stimulation [22]. ELKS, an essential regulatory subunit of the IKK complex, recruits Ikappa-B-alpha to the IKK complex and regulates IKK activation [23].ATM is also exported in a NEMO-dependent manner to the cytoplasm, where it promotes IKK activation in an ELKS-dependent manner [24]. ATM- and NEMO-dependent ubiquitination of ELKS results in ubiquitin-dependent assembly of the TAK1/TAB2/3 and NEMO/IKK complexes, and IKK and NF-κB activation in response togenotoxic stimuli [25]. SDCCAG8 was found to interact with centriolar satellite proteins (OFD1 and AZI1), members of the endosomal sorting complex (RABEP2 and ERC1), and with non-muscle myosin motor proteins (MYH9, MYH10, and MYH14) at the centrosome [26]. Mutations in ERC1 likely impact centrosome function. ERC1-RET fusions resulting from a translocation, t(10;12)(q11;p13), have also been observed in thyroid papillary carcinoma [27, 28].

Voltage-gated potassium (Kv) channels are the most structurally and functionally complex class of voltage-gated ion channels. These channels have multiple functions including regulation of neurotransmitter release, heart rate, insulin secretion, neuronal excitability, epithelial electrolyte transport, smooth muscle contraction, and cell volume. *KCNG4* encodes a member of the potassium channel, voltage-gated, subfamily G and functions as a modulatory subunit. Multiple alternatively spliced variants have been observed in normal and malignant tissue. Sequence analysis indicated Kv6.3 was a previously uncharacterized member of the Kv6 subfamily. The other splice variants were the first members of two unique subfamilies, Kv10.1 and Kv11.1. These channels did not produce K+ currents when expressed in mammalian cells, although they have all of the hallmarks of voltage-gated K+ channel subunits. Kv6.3, Kv10.1, and Kv11.1 alone did not localize to the plasma membrane, but were retained in the endoplasmic reticulum [29]. Muller et al. found that Dlk1 suppressed Notch signaling and induced expression of the K(+) channel subunit *KCNG4* to modulate delayed rectifier currents [30]. This gene may affect the oocyte during the second meiotic division.

Sporadic CHM with androgenetic origin results from fertilization of an empty oocyte with paternal genes. But the precise mechanism underlying the empty oocyte is currently unclear. According to our results, *ERC1* and *KCNG4* are highly conserved genes associated with centrosome function. ERC1 interacts with SDCCAG8, a major component of the centriole. KCNG4 may impact centrosome function by modulating the K^+^ current. We hypothesize that *ERC1*c.G48C(p.Q16H) and *KCNG4* c.G1114A(p.G372S) may play a role in the generation of an empty oocyte during the second meiotic division by altering centrosome function. However, there are no reliable methods to investigate empty oocytes *in vitro* or *in vivo*. This is partly because CHM have not been observed in animal models. However, a few partial hydatidiform moles have been described in cats and Friesian cows [31, 32].

We identified two single nucleotide polymorphisms(SNPs)in *ERC1*and *KCNG4* that were associated with an increased risk of CHM. Women with these two variants should be carefully monitored. A pathological examination of the product of pregnancy in women with an inevitable abortion should be performed. Additional studies with larger sample sizes and long-term follow-up are necessary. Functional studies of ERC1 and KCNG4 in animal models of CHM are also required in order to clarify the pathogenesis of CHM.

## MATERIALS AND METHODS

### Study population and sample preparation

We analyzed 250 Chinese Han women with non-familial CHM (16–56 years old) and 652 age-matched healthy controls (21–45 years old) who were treated at several tertiary centers across China between November 2011 and July 2015. The meanage ± standard deviation (SD) of the CHM patients and controls was 31.6±9.3 and 29.1±4.6 years, respectively. All subjects provided written consent. Clinical data was collected by chart review and self-reporting. CHM was histologically confirmed by a gynecological pathologist (B.L.) according to the consensus diagnostic criteria (WHO 2014). Women with familial, recurrent CHM were excluded from the study by chart review and follow-up visit. Healthy controls were defined as Han women who had at least one previous normal delivery of a healthy baby, no history of an abortion (including spontaneous and artificial) or abnormal pregnancy, and no family history of cancer or gestational trophoblastic disease. Each subject provided approximately 15mLof peripheral blood, which was stored in EDTA-anticoagulant tubes at -80°C until use. Genomic DNA was extracted using the QiagenQIAamp DNA Blood Mini Kit according to the manufacturer’s instructions. Genomic DNA was stored in Tris-EDTA buffer at -80°C until use.

### Whole-exome sequencing and data analysis

Whole-exome sequencing was performed on genomic DNA from 51 patients and 47 controls (the initial screening group). Whole-exome capture libraries were constructed from approximately 3μg of purified genomic DNA following fragmentation, end repair, phosphorylation, and ligation. Ligated samples were hybridized to the exon capture arrays using Roche NimbleGen V2 (44.1 Mbp). Captured DNA was annealed at 95°C and single-stranded DNA amplified and sequenced on an Illumina HiSeq2000.

Exome sequencing data were analyzed using the Exome Analysis Pipeline (DNAnexus, http://www.dnanexus.com). Variant detection and genotyping were performed on exomes and the flanking 500 bp downstream of the 5’- or 3’-UTRs. A BAM (binary alignment map) file for each sample was generated from the alignment of the Illumina sequence reads to the human genome (hg19). SNV calls were evaluated and each variant annotated.

### Mass spectrometry

We selected 168 rare SNPs that were identified by whole-exome sequencing and were predicted to be deleterious. We used the Sequenom MassARRAY technology platform with the iPLEX GOLD chemistry [33, 34] as a second-round filter. An additional 199 patients and 400 controls were tested. Genotyping analysis was performed using the commercial iPLEX Gold SNP genotyping kit and the MassARRAY platform according to the manufacturer’s protocols. Mass determination was done with the MALDI-TOF mass spectrometer. The MassARRAY Typer 4.0 software was used for data acquisition. SNP genotypes were called after cluster analysis using the default setting. Genotype calls were reviewed manually to correct potential clustering artifacts. Assays with less than a 90% call rate within the same SpectroCHIP were excluded from further analysis.

### Validation of selected candidate variant sites by Sanger sequencing

Two SNPs were selected for further validation by Sanger sequencing in 250 cases and 652 controls, including an additional 205 controls. Both non-synonymous SNPs were believed to be significant based on mass spectrometry. The primer sequences are shown in Supplementary Table 1. Genomic DNA was amplified by PCR and sequenced using an ABI 3730XL DNA analyzer.

### Statistical analysis

Statistical analysis was performed using SPSS 20.0 (SPSS, Inc., Chicago, IL, USA). Fisher’s exact tests and Chi-square tests were used to evaluate the associations between CHM and single-variant genes. The ORs and 95% confidence intervals(CIs) were calculated to estimate the relative risk of CHM. AssotesteR was used to detect the association between CHM and multi-variant genes. AssotesteR is a statistical package for R that is commonly used in genetic association studies of rare variants and binary (dichotomous) traits (https://cran.r-project.org/web/packages/AssotesteR/index.html). The threshold for statistical significance was one-sided p-values of 0.05, assuming that rare alleles increase the risk of CHM.

## SUPPLEMENTARY MATERIALS TABLES












