# Supplementary material for: Whole-exome sequencing reveals genetic variants in ERC1 and KCNG4 associated with complete hydatidiform mole in Chinese Han women
Source: Oncotarget. 2017 Sep 8;8(43):75264–71. doi: 10.18632/oncotarget.20769 (PMC5650418; doi:10.18632/oncotarget.20769)
Supplement: Supplementary file 1 [file oncotarget-08-75264-s001.pdf]

Whole-exome sequencing reveals genetic variants in ERC1 and KCNG4 associated with complete hydatidiform mole in Chinese Han women

SUPPLEMENTARY MATERIALS

Supplementary Table 1: The primers for PCR amplification of candidate SNPs

Sites			Sequence
ERC1	c.G48C(p.Q16H)	up	AGATACTTCTTCAAGATTGGACAGC
		down	GCAGTCATCCGAACACCGTAAGG
KCNG4	c.G1114A(p.G372S)	up	AGGTAGGAGTGGGAGAAGGTGT
		down	TCGCTGGCGGTGTCTGAGGAG

The PCR condition: annealing temperature is 50°C and amplification with 25 cycles.

Supplementary Table 2: Summary of the exome sequencing data for 98 samples

See Supplementary File 1

Supplementary Table 3: List of potentially significant SNVs between CHM patients and healthy controls

See Supplementary File 2

Supplementary Table 4: The distribution of SNVs identified using MassARRAY technology

See Supplementary File 3

Supplementary Table 5: Possible genes that influence the risk of CHM

See Supplementary File 4

Supplementary Table 6: Comparison between WES, mass spectrometry, and Sanger sequencing

See Supplementary File 5
