# Supplementary material for: Whole-exome sequencing reveals genetic variants in ERC1 and KCNG4 associated with complete hydatidiform mole in Chinese Han women
Source: Oncotarget. 2017 Sep 8;8(43):75264–71. doi: 10.18632/oncotarget.20769 (PMC5650418; doi:10.18632/oncotarget.20769)
Supplement: Supplementary file 2 [file oncotarget-08-75264-s002.doc]

**S2** The summary data of exome sequencing from 100 samples

| **Samples_Ot** | **Coverage** | **Yield(bp)** | **Total_reads** | **Mapped_reads** |
| --- | --- | --- | --- | --- |
| Ot2212 | 63 | 7,201,513,200 | 72,015,132 | 65,886,745 |
| Ot2213 | 67 | 6,898,579,200 | 68,985,792 | 63,269,582 |
| Ot2214 | 55 | 5,389,443,800 | 53,894,438 | 49,490,352 |
| Ot2215 | 62 | 6,408,802,600 | 64,088,026 | 58,543,386 |
| Ot2216 | 58 | 6,193,422,200 | 61,934,222 | 57,284,831 |
| Ot2217 | 66 | 7,102,900,200 | 71,029,002 | 65,346,636 |
| Ot2218 | 52 | 5,902,437,600 | 59,024,376 | 54,378,608 |
| Ot2219 | 58 | 6,143,117,200 | 61,431,172 | 56,667,629 |
| Ot2220 | 107 | 11,697,215,400 | 116,972,154 | 107,920,174 |
| Ot2221 | 66 | 6,998,674,800 | 69,986,748 | 65,503,556 |
| Ot2222 | 60 | 5,703,057,600 | 57,030,576 | 53,620,471 |
| Ot2223 | 61 | 6,897,196,000 | 68,971,960 | 63,057,578 |
| Ot2224 | 73 | 7,733,606,600 | 77,336,066 | 69,519,680 |
| Ot2225 | 73 | 8,058,607,000 | 80,586,070 | 72,335,690 |
| Ot2226 | 78 | 8,187,343,800 | 81,873,438 | 74,089,001 |
| Ot2227 | 79 | 8,420,236,400 | 84,202,364 | 75,459,824 |
| Ot2228 | 86 | 8,826,684,600 | 88,266,846 | 81,259,661 |
| Ot2229 | 85 | 8,046,380,400 | 80,463,804 | 74,706,436 |
| Ot2230 | 86 | 8,181,497,400 | 81,814,974 | 76,567,986 |
| Ot2231 | 74 | 7,278,523,800 | 72,785,238 | 68,189,015 |
| Ot2232 | 66 | 8,287,553,400 | 82,875,534 | 76,338,864 |
| Ot2233 | 70 | 7,535,030,200 | 75,350,302 | 70,567,565 |
| Ot2234 | 70 | 7,171,764,400 | 71,717,644 | 67,304,577 |
| Ot2235 | 71 | 8,370,534,200 | 83,705,342 | 74,217,140 |
| Ot2236 | 60 | 8,093,197,600 | 80,931,976 | 71,299,197 |
| Ot2237 | 67 | 8,067,272,400 | 80,672,724 | 71,698,627 |
| Ot2238 | 57 | 7,399,430,600 | 73,994,306 | 65,632,480 |
| Ot2239 | 54 | 7,331,866,800 | 73,318,668 | 65,316,354 |
| Ot2240 | 71 | 8,619,928,200 | 86,199,282 | 76,255,714 |
| Ot2241 | 70 | 8,710,277,400 | 87,102,774 | 77,166,611 |
| Ot2242 | 79 | 9,583,309,400 | 95,833,094 | 84,925,243 |
| Ot2243 | 72 | 8,084,666,800 | 80,846,668 | 71,482,128 |
| Ot2245 | 78 | 8,018,205,000 | 80,182,050 | 71,251,102 |
| Ot2246 | 64 | 7,622,445,400 | 76,224,454 | 66,757,296 |
| Ot2247 | 67 | 7,283,804,200 | 72,838,042 | 63,863,084 |
| Ot2248 | 59 | 6,163,706,200 | 61,637,062 | 54,800,888 |
| Ot2249 | 75 | 8,499,198,800 | 84,991,988 | 74,734,437 |
| Ot2251 | 65 | 8,159,862,200 | 81,598,622 | 70,939,814 |
| Ot2252 | 72 | 7,693,179,400 | 76,931,794 | 68,509,273 |
| Ot2253 | 58 | 6,977,438,600 | 69,774,386 | 64,299,955 |
| Ot2254 | 62 | 6,695,824,200 | 66,958,242 | 61,699,218 |
| Ot2255 | 52 | 5,993,935,200 | 59,939,352 | 55,089,012 |
| Ot2256 | 50 | 5,707,814,800 | 57,078,148 | 52,298,572 |
| Ot2257 | 62 | 6,738,360,600 | 67,383,606 | 61,068,562 |
| Ot2258 | 64 | 6,277,379,000 | 62,773,790 | 58,424,656 |
| Ot2259 | 55 | 6,365,613,400 | 63,656,134 | 58,279,841 |
| Ot2260 | 59 | 6,050,196,200 | 60,501,962 | 56,363,495 |
| Ot2262 | 64 | 6,134,338,800 | 61,343,388 | 56,795,770 |
| Ot2264 | 74 | 7,620,220,600 | 76,202,206 | 69,985,026 |
| Ot2265 | 58 | 6,111,564,000 | 61,115,640 | 56,462,353 |
| Ot2266 | 59 | 6,154,089,600 | 61,540,896 | 57,081,109 |
| Ot2267 | 61 | 6,250,962,800 | 62,509,628 | 57,785,179 |
| Ot2268 | 59 | 6,171,917,200 | 61,719,172 | 57,100,741 |
| Ot2269 | 78 | 7,710,631,400 | 77,106,314 | 71,568,797 |
| Ot2270 | 67 | 7,083,948,400 | 70,839,484 | 65,069,798 |
| Ot2271 | 82 | 8,031,454,800 | 80,314,548 | 73,661,040 |
| Ot2272 | 69 | 6,855,525,200 | 68,555,252 | 62,561,954 |
| Ot2273 | 65 | 5,876,329,600 | 58,763,296 | 54,008,489 |
| Ot2274 | 72 | 7,227,458,200 | 72,274,582 | 65,156,502 |
| Ot2275 | 73 | 7,290,331,000 | 72,903,310 | 68,411,061 |
| Ot2276 | 72 | 6,900,522,800 | 69,005,228 | 62,748,443 |
| Ot2277 | 73 | 7,509,990,200 | 75,099,902 | 68,173,562 |
| Ot2278 | 66 | 6,308,975,200 | 63,089,752 | 56,581,084 |
| Ot2279 | 59 | 6,215,114,200 | 62,151,142 | 56,176,042 |
| Ot2280 | 78 | 7,553,594,800 | 75,535,948 | 71,235,927 |
| Ot2281 | 67 | 6,192,994,800 | 61,929,948 | 58,513,564 |
| Ot2282 | 62 | 5,862,767,400 | 58,627,674 | 55,338,160 |
| Ot2283 | 81 | 8,101,539,800 | 81,015,398 | 76,357,840 |
| Ot2284 | 79 | 7,677,740,200 | 76,777,402 | 72,819,169 |
| Ot2285 | 78 | 8,097,940,400 | 80,979,404 | 76,300,728 |
| Ot2286 | 57 | 6,296,529,200 | 62,965,292 | 56,709,981 |
| Ot2287 | 74 | 8,036,837,200 | 80,368,372 | 72,023,545 |
| Ot2288 | 77 | 8,136,721,400 | 81,367,214 | 72,327,798 |
| Ot2289 | 80 | 8,391,038,400 | 83,910,384 | 74,543,445 |
| Ot2290 | 79 | 8,054,619,800 | 80,546,198 | 71,123,917 |
| Ot2291 | 81 | 8,360,648,400 | 83,606,484 | 75,116,567 |
| Ot2454 | 62 | 7,695,931,800 | 76,959,318 | 66,891,026 |
| Ot2455 | 52 | 6,371,330,600 | 63,713,306 | 54,991,984 |
| Ot2456 | 61 | 7,059,644,200 | 70,596,442 | 61,202,056 |
| Ot2457 | 58 | 7,350,143,600 | 73,501,436 | 63,595,972 |
| Ot2458 | 59 | 7,193,564,400 | 71,935,644 | 61,991,150 |
| Ot2459 | 59 | 7,415,508,000 | 74,155,080 | 64,488,213 |
| Ot2460 | 57 | 6,370,602,800 | 63,706,028 | 56,277,072 |
| Ot2462 | 58 | 6,156,860,000 | 61,568,600 | 54,351,356 |
| Ot2464 | 64 | 7,211,333,200 | 72,113,332 | 67,107,767 |
| Ot2465 | 65 | 7,095,147,800 | 70,951,478 | 65,879,981 |
| Ot2466 | 64 | 7,749,332,000 | 77,493,320 | 71,579,158 |
| Ot2467 | 75 | 7,924,285,800 | 79,242,858 | 74,027,270 |
| Ot2468 | 67 | 7,464,950,800 | 74,649,508 | 68,306,357 |
| Ot2469 | 65 | 5,983,416,000 | 59,834,160 | 56,321,488 |
| Ot2470 | 59 | 5,787,542,000 | 57,875,420 | 53,857,882 |
| Ot2471 | 60 | 5,619,764,000 | 56,197,640 | 52,833,152 |
| Ot2472 | 67 | 6,459,026,800 | 64,590,268 | 60,635,601 |
| Ot2473 | 57 | 6,342,741,400 | 63,427,414 | 58,774,065 |
| Ot2261 | 78 | 7,341,545,800 | 73,415,458 | 67,942,704 |
| Ot2263 | 67 | 7,019,203,600 | 70,192,036 | 64,558,718 |
| Ot2461 | 65 | 6,877,891,200 | 68,778,912 | 60,568,967 |
| Ot2463 | 52 | 5,366,975,176 | 53,872,892 | 47,652,846 |
